# Conservation perspectives of small-scale private forest owners in Europe: A systematic review

**DOI:** 10.1007/s13280-021-01615-w

**Published:** 2021-09-20

**Authors:** Malin Tiebel, Andreas Mölder, Tobias Plieninger

**Affiliations:** 1 Department of Agricultural Economics and Rural Development, University of Göttingen, Platz der Göttinger Sieben 5, 37073 Göttingen, Germany; 2 Department of Forest Nature Conservation, Northwest German Forest Research Institute (NW-FVA), Prof.-Oelkers-Straße 6, 34346 Hann. Münden, Germany; 3 Faculty of Organic Agricultural Sciences, University of Kassel, Steinstraße 19, 37213 Witzenhausen, Germany

**Keywords:** Integrative forest management, Multifunctional forestry, Nature conservation, Small-scale forestry, Sustainable forest management, Systematic literature review

## Abstract

Private forest owners are the main forest ownership group within Europe, and important conservation values have been found on their land. Yet, small plot sizes, societal heterogeneity, and structural changes impede developing and implementing effective conservation programs in private forests. We present a systematic literature review focusing on small-scale private forest owners and their perspectives on nature conservation by synthesizing research approaches, social-ecological drivers, and policy recommendations. Conservation perspectives were positively related to female gender, higher levels of education, formalized forest management, an active relation to the forest, and ecological values of the property. In contrast, high age, rural orientation, economic forest management factors, large parcel size, and economic and sentimental property values negatively influenced conservation perspectives. Applying a natural resource conflict management framework, we synthesized recommendations covering three dimensions: substance, procedure, relationship. Considering perspectives of small-scale private forest owners in current forestry decision-making has great potential to strengthen sustainable forest management that integrates nature conservation and resource use.

**Supplementary Information:**

The online version contains supplementary material available at 10.1007/s13280-021-01615-w.

## Introduction

While forests are crucially important in sustaining biodiversity and human wellbeing (European Commission 2013), current forest management in Europe is facing multiple challenges. Forests are increasingly vulnerable due to climate change effects (Seidl et al. 2007) such as more frequent storms and droughts (Lindner et al. 2010) or bark-beetle infestations (Jakoby et al. 2019). At the same time, often contradictory societal demands exist which range from instrumental to intrinsic and social objectives and thus include timber production, biodiversity conservation, and recreation (Sandström et al. 2016). As a consequence, many countries have developed programs for integrative multifunctional forest management in publicly owned forests (Borrass et al. 2017) or ensured a certain degree of state intervention (Wilkes-Allemann and Lieberherr 2020). However, private forest owners are also important stakeholders to be considered when aspiring efficient biodiversity conservation (Mayer 2019) as they represent the largest forest ownership class in Europe. Within the European Union, 60% of forest land is privately owned (European Commission 2013), ranging from 11% in Bulgaria to 93% in Portugal (Zivojinovic et al. 2015). The share of private forest holdings increased by 18% from 1990 to 2010, mainly due to privatization and restitution of forest land in former socialist countries (Forest Europe 2015; Weiss et al. 2019). Large private forests, either owned by individuals, industrial companies, or institutions like churches, can reach sizes between a few hundred and several thousand hectares. Small-scale private forests, at the other end of the size range, are frequently much smaller than ten hectares. Also known as non-industrial, smallholder, or family forests, they are abundant in most European countries (UNECE and FAO 2020).

Small-scale private forests can harbour important conservation values (Mölder et al. 2021). For example, small private forests in southwest Germany had more pronounced structural diversity, higher amounts of deadwood, as well as a larger capacity to store carbon than state- and municipality-owned forests (Schaich and Plieninger 2013). The same ownership type also showed a higher density and diversity of tree-related microhabitats (Johann and Schaich 2016). Similarly, small private forests in Latvia exhibited higher tree species richness and a more complex vertical canopy structure (Rendenieks et al. 2015). Patch heterogeneity and quality were the main predictors of plant species richness in northeast German small private forests. The observed variability in stand structures and species assemblages resulted from the history of private ownership and its smallholder social structures (Wulf and Kolk 2014). Furthermore, small private forests are known to harbour structural relicts of historical forest management techniques such as coppicing which provide valuable habitats (Mölder 2016). Despite the frequent occurrence of conservation values in small private forests, the implementation of appropriate conservation measures is conflict-prone. In particular, disputes between forest owners and the public can occur when regulations regarding biodiversity conservation or recreation schemes are conflicting with landowners’ rights or management targets (Bergstén et al. 2018). Reasons for a dispute can be a lack of communication (Brukas et al. 2018), fear of economic consequences when restrictions reduce the income from forestry (Widman 2015), the level of compensation (Götmark 2009), loss of decision power (Jokinen et al. 2018), limitations of property rights, past negative experiences (Widman 2015), as well as high administrative burdens, for example regarding grant applications (Urquhart et al. 2012). Conservation policies need to not only be effective in an ecological sense, but also be accepted locally (Jokinen et al. 2018). Thus, an understanding of forest owners’ motivations (Mitani and Lindhjem 2015), values (Nordén et al. 2017), attitudes, and behaviors (Butler et al. 2016), as well as of behavior-influencing drivers (Gatto et al. 2019) is crucial to mitigate these conflicts and to provide decision support for effective conservation and forest management.

Another challenge for implementing successful nature conservation measures is the small-sized structure of private forest ownership. A comparison of nine European countries revealed that 61% of these estates are smaller than one hectare (Schmithüsen and Hirsch 2010). In Germany for instance, private forest holdings have an average size of 12 ha (Feil et al. 2018). Furthermore, it is necessary to consider the large individual heterogeneity amongst small-scale forest owners that translates into a variety of values (Richnau et al. 2013), motivations, objectives (Urquhart and Courtney 2011), and management strategies (Haugen 2016). This diversity is increasing (Bergstén et al. 2018; Joa and Schraml 2020) with societal change and particularly with new owners or heirs often having diverging characteristics (Nordlund and Westin 2011; Häyrinen et al. 2015). Both urban and non-resident forest owners are strongly on the rise (Nordlund and Westin 2011; Eggers et al. 2014; Juutinen et al. 2020). At the same time, the proportions of farmers (Eggers et al. 2014) and people experienced in forestry and forest management practices (Urquhart and Courtney 2011) are decreasing. Furthermore, a more diverse set of objectives including nature conservation can be identified (Häyrinen et al. 2015), while financial dependence on forestry is diminishing (Eggers et al. 2014). Due to these socio-structural changes, appropriate research is needed to evaluate and design effective nature conservation policies.

Various studies have focused on determining factors that influence small-scale private forest owners’ attitudes (Bieling and Schraml 2004; Bergseng and Vatn 2009; Feliciano et al. 2017; Danley 2019) or behaviors (Korhonen et al. 2013; Widman 2015; Vainio et al. 2018) towards nature conservation in Europe. However, most of these studies have been carried out at a regional level. A systematic review of such studies offers the opportunity to aggregate this regional knowledge at the European level and allows for the identification of cross-local patterns and trends of the factors influencing conservation perspectives. Therefore, this study will summarize research approaches to reveal methodological research gaps in this field. Moreover, we assess social-ecological drivers influencing conservation perspectives. This enables us to identify particular patterns amongst the heterogeneous group of small-scale private forest owners. Finally, we synthesize policy recommendations to provide an overview for decision-makers. Thus, we raise the following research questions:

- Which approaches have been applied to investigate small-scale private forest owners and their interactions with nature conservation?
- Which social-ecological factors influence small-scale private forest owners’ conservation perspectives?
- Which recommendations regarding conservation policies targeting small-scale private forest owners have been derived?

## Methods

### Search strategy

We applied the Preferred Reporting Items for Systematic Reviews and Meta-Analyses (PRISMA) standards to perform a transparent and complete systematic review (Moher et al. 2009). Our search strategy consisted of four categories (Table 1). Each term of each category was separated by “OR”, while categories were separated by “AND”, which led to results including at least one term of each category in their title, abstract, or keywords. Our review covered scientific publications that were indexed in the following databases: Web of Science, Scopus, and Forest Science Database. We restricted our search to articles being published between 2004 and May 2021 and retrieved 5829 publications in total. After the removal of duplicates, 5101 articles remained.

**Table 1:** Applied search terms for the literature review

Scale terms	Ecosystem terms	Nature conservation terms	Management and motivation terms
smallholder*	woodland*	biodiv*	behavior*
small-scale	forest*	conserv*	behaviour*
smallscale		habitat*	decision*
family		wildlife*	manag*
private*		close-to-nature	action*
non-industrial		deadwood	intention*
		dead wood*	motiv*
		multifunction*	value*

In the next step, we screened these articles and tested them for their eligibility. We considered those studies relevant that were conducted within the European Union or Switzerland/Norway/Great Britain. Eligible studies had to identify the perspectives of small-scale private forest owners on nature conservation. Only studies based on empirical data were included. We applied these criteria in three rounds by screening the titles (450 remaining articles), the abstracts (139 remaining articles), and the full texts of the remaining articles (41 remaining articles). We excluded most articles in the process of title screening as they focused either on other geographical regions or on ecological rather than social parameters. We complemented our strategy by including eligible literature found in the references of the retrieved articles and due to recommendations of colleagues. Our final sample comprised 46 relevant publications, further referred to as “core studies”.

### Data extraction and analysis

We formulated rules regarding data extraction a-priori and continuously revised and extended them (Pullin and Stewart 2006). Spreadsheets were used to extract data, thereby gathering information on general characteristics of each study such as year of publication, journal, spatial scale, sample size, reply rate, average size of the forest holdings, sampling of other stakeholders, research approach, and thematic core areas.

Furthermore, we recorded variables influencing three thematic core areas (attitude, conservation decisions, pro-conservation ownership) and categorized them according to Gatto et al. (2019). Henceforth, we use the term “conservation perspective” to refer to these thematic core areas. Parameters affecting the process and thus a fourth thematic core area were not included as positions towards certain elements of a process such as compensation differ from conservation perspectives. Moreover, we included only categories, whose variables were considered by at least 15% of the studies. We distinguished three directions of influence: positive, negative, and ambiguous. For quantitative results, we considered positive or negative affects only when being significant (p ≤ 0.05), while we recorded non-significant influences as ambiguous. Ambiguous outcomes also included contradictory results within one article as well as variables that did not show any influence. Regarding qualitative outcomes, we recorded positive or negative influences when these were described as such by the authors of the respective study. To account for this difference in research design, we introduced a strength of evidence measure, ranging from 1 (poor: An author’s statement cannot be directly traced back neither to qualitative nor to quantitative data. It is unclear how the author has derived his/her conclusion.) to 5 (very good: The data and its significance in respect of a certain parameter are explicitly presented within a core study.).

Additionally, we collected policy recommendations given within the core studies. Based on the assumption that recommendations aim to improve conflicting situations, they were categorized according to the natural resource conflict management framework by Walker and Daniels (1997) into the three dimensions 1) substance, 2) procedure, and 3) relationship. This model aims to foster the comprehension of a conflict. Its three-dimensional structure illustrates the interrelation between the dimensions (Walker and Daniels 1997). This framework is also referred to as conflict management progress triangle and has previously been used to analyse European forest policy (Edwards and Kleinschmit 2013) and the conflict between conservation and other forest-related interests (Niemelä et al. 2005).

## Results

### Research approaches

**Fig. 1:**
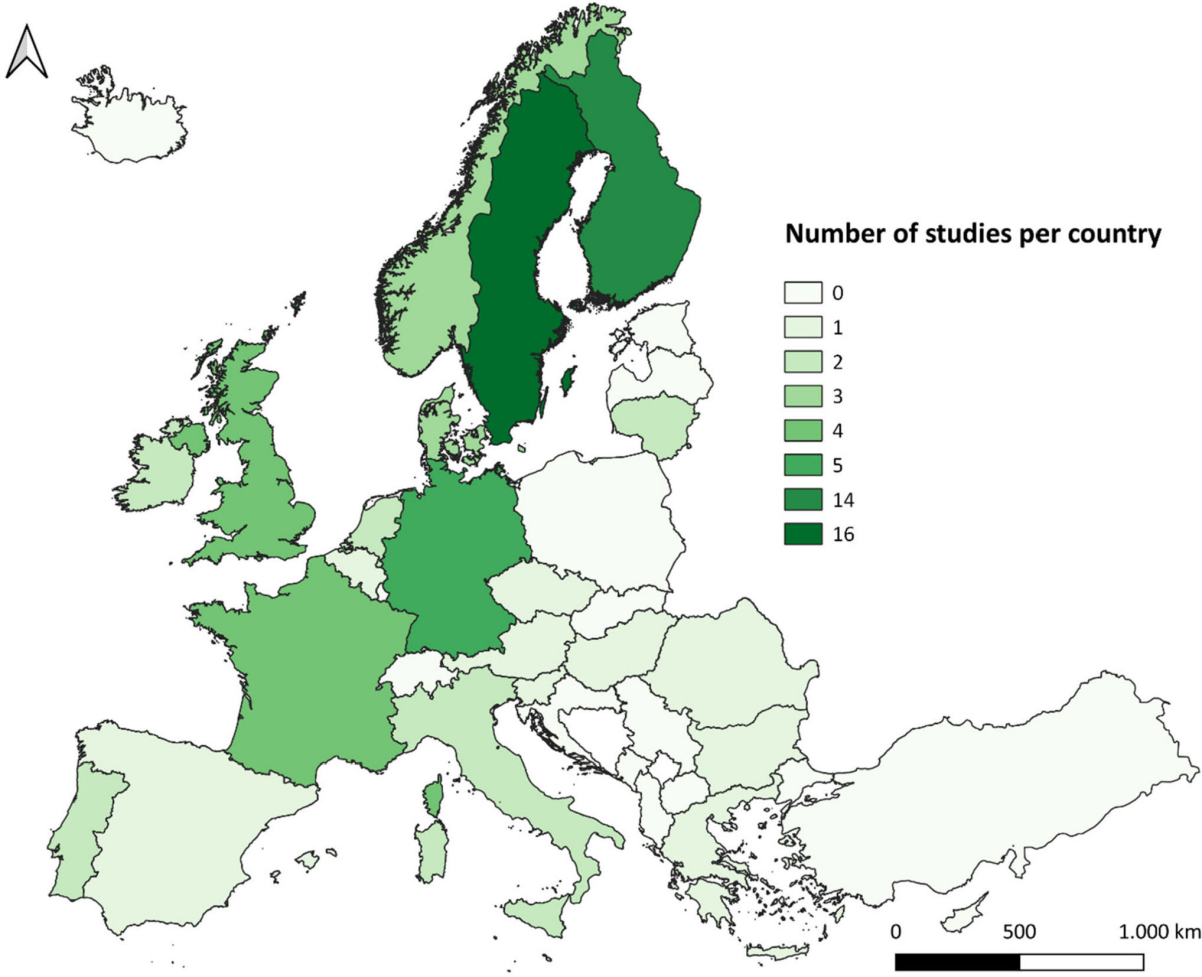
Spatial focus of the core studies (*n* = 46). Administrative boundaries: © EuroGeographics

The 46 core studies were conducted in 22 countries, whereby four studies were located in more than one country. Eight countries were only considered once (Austria, Bulgaria, Czech Republic, Greece, Hungary, Romania, Slovenia, Spain) and had been included in studies focusing on multiple countries. Sweden and Finland were included as case studies most often, by 16 and 14 studies, respectively (Fig. 1).

**Fig. 2:**
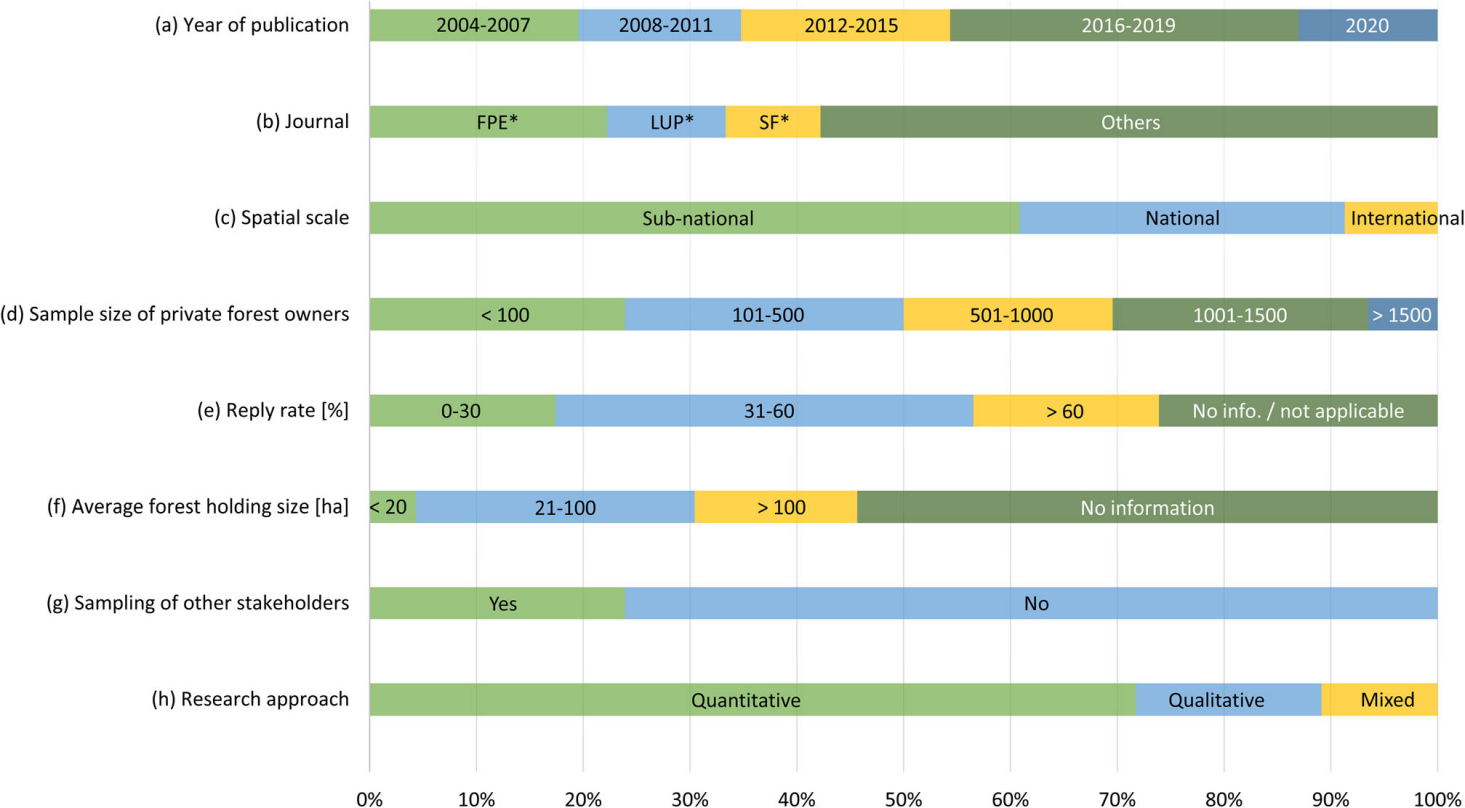
General characteristics and research approaches of the 40 core studies. *FPE Forest Policy and Economics, *LUP* Land Use Policy, *SF*  Small-scale Forestry

The core studies evenly spread across three time periods with 15–20% being published between 2004–2007, 2008–2011, and 2012–2015. Forty-six percent of all studies were released from 2016 onward (Fig. 2a). The most frequent journals (42%) were Forest Policy and Economics (22%), Land Use Policy (11%), as well as Small-scale Forestry (9%, Fig. 2b). A majority of core studies focused on a sub-national sample (61%), while 30% were based on a national sample. A minority of 9% had an international scope (Fig. 2c). The sample sizes varied widely from 11 (Van Gossum et al. 2011) to 2398 (Nordén et al. 2017) forest owners. Twenty-four percent of the core studies included < 100 forest owners, 26% inquired 101–500, 20% 501–1000, and 24% asked 1001–1500 forest owners. Seven percent of studies used data of more than 1500 forest owners (Fig. 2d). Thereby the response rates ranged from 20% (Vedel et al. 2015) to 81% (Boon and Meilby 2007; Urquhart 2009; Urquhart and Courtney 2011), with the biggest share of such studies (39%) having a response rate in the range of 31 to 60%. Twenty-six percent of studies did not report this figure (Fig. 2e). Understandings of “small-scale forest ownership” varied, with reported, calculated, or inquired average forest holding sizes ranging from 9 (Bieling 2004) to 251 ha (Brukas et al. 2018). The majority of the studies (54%) did not report the average holding size (Fig. 2f). While most studies only focused on data originating from forest owners, 24% also sampled other stakeholder groups such as forest administration (Widman 2015), nature conservation organizations, forest industry (Hysing and Olsson 2005), tourism actors (Hallikainen et al. 2010), or the general public (Nordén et al. 2017) (Fig. 2g). Most often, quantitative methods were used to analyse the data (72%, Fig. 2h).

Further, we identified four thematic core areas on which the studies concentrate: attitude, conservation decisions, pro-conservation ownership, and process. Most studies (70%) included an analysis of attitudes either towards forest conservation in general (Bergseng and Vatn 2009), and towards certain elements such as maintaining a shrub layer (Van Gossum et al. 2005), or they concentrated on the willingness to act in a pro-conservation way in the future. This included, for example, the willingness to carry out more environmental measures prospectively (Danley 2019). A share of 46% of the studies focused on conservation decisions, summarizing studies which analyse choices such as managing the forest according to close-to-nature principles (Bieling 2004) or participating in conservation programs (Primmer et al. 2014; Vainio et al. 2018). An equal share of 46% of all core studies focused on the conservation process and thus included research on attitudes towards the process itself (Jokinen et al. 2018) or towards certain aspects such as compensation (Lindhjem and Mitani 2012). About 24% of the studies used typologies to classify forest owners and thereby included a category called “conservationists” (Hallikainen et al. 2010) or similar (Deuffic et al. 2018). Fifty-nine percent of the core studies concentrated on more than one of these thematic core areas.

### Social-ecological drivers

The evaluation of the owners’ objective and subjective factors (Table 2a) revealed that female gender, higher levels of education, a certain degree of formalized forest management, and an active relation to the forest had a positive influence on conservation perspectives. For instance, being a member of an ownership association increased the likelihood of having a certified forest property in Sweden (Danley 2019). On the contrary, age and a rural orientation had a rather negative affect. To give an example, aesthetic and conservation objectives were important for female, urban-oriented, as well as highly educated owners (Häyrinen et al. 2015). Similarly, owners from the environmental/recreational ownership cluster were characterized by an urban orientation as well as a high level of activity (Boon and Meilby 2007). In the forest management category (Table 2b), economic factors had a weakening influence on conservation perspectives. 90% of Finish forest owners who commercially manage their land did, for instance, not have conservation interest (Paloniemi and Tikka 2008). Product factors had an equal degree of influence in both directions. Regarding structural factors (Table 2c), a large forest holding size negatively affected conservation perspectives whereas ecological value had a positive influence. In Germany, for instance, the appreciation for coniferous stands increased with the size of the forest holding, while it decreased with a higher share of deciduous trees at the own property (Bieling and Schraml 2004). Perceived economic as well as sentimental values (Table 2d) had a negative influence. For instance, forest owners classified as conservationists had a short duration of ownership, a small rate of acquisition by inheritance, and the lowest share of income from the forest (Ingemarson et al. 2006). Almost all parameters have been found to feature both a positive and negative influence.

**Table 2:**
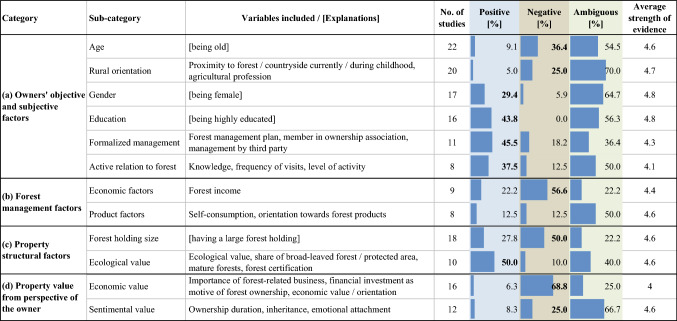
Effects of social-ecological factors on conservation perspectives, categorized according to Gatto et al. (2019). Bold numbers show the main direction of influence. The strength of evidence measure ranges from 1 (poor) to 5 (very good)

Regarding all factors, the average strength of evidence was larger than or equal to 4.0 with gender and education (Table 2a) showing the highest value (4.8). In contrast, the economic property value (Table 2c) had an average strength of evidence of 4.0 and thus, showed the weakest strength of evidence.

### Policy recommendations

Most studies (89%) provided recommendations focusing on the substance of conservation policy (Table 3a). Here, suggestions for improving or implementing certain instruments spread across 19 different measures (recommended by 83%). The recommendations also included critical comments. For example, certification was contested due to legitimacy problems (Hysing and Olsson 2005) and missing proof of its ecological benefits (Danley 2019). Payments-for-ecosystem-services and incentive schemes were criticized due to the little additionality gained as forest owners often apply for measures they implement anyway (Urquhart et al. 2012; Vedel et al. 2015). In general, the studies expressed the need for increased attractiveness of the instruments (recommended by 57%), for example via the establishment of suitable conditions. Conservation programs should be fair (Joa and Schraml 2020), have acceptable conditions (Mäntymaa et al. 2009), and be broadly based, for example by including economic and recreational aspects alongside ecological issues. A combination of different conservation instruments was considered useful (Van Gossum et al. 2005). At the same time, studies pointed out the need to provide sufficient resources, for example to ensure a functioning monitoring system (Widman 2015; Brukas et al. 2018). Further, studies called for paying more attention to the heterogeneity amongst forest owner types (Wiersum et al. 2005; Bergstén et al. 2018; Brukas et al. 2018; Pynnönen et al. 2018; Joa and Schraml 2020) and their ownership rights (Polomé 2016; Bostedt et al. 2019; Joa and Schraml 2020).

**Table 3:** Policy recommendations categorized according to the natural resource conflict management framework by Walker and Daniels (1997). Percentages (rounded to full numbers) show the share of studies with recommendations within a certain category

Recommendation category	Sub-category	Details
(a) Substance *Design of policy instruments *(89%)	Recommendations towards certain instruments (83%)	Voluntary instruments (35%), compensation (22%), incentives (20%), certification (17%), monitoring (13%), payment for ecosystem services, management plans (each 9%), rewards, regulations, market mechanisms, new policy approaches (each 7%), others (22%)
	Increase the attractiveness of instruments (57%)	Suitable conditions (30%), consideration of heterogeneity of forest owners (20%), consideration of ownership rights (13%), others (4%)
(b) Procedure *Process and implementation of policy instruments* (65%)	Improvement of information distribution (59%)	Increase information distribution (30%), advisory services (28%), focus on other relevant stakeholders (22%), peer-learning and regional demonstration, educational programs, (each 20%), include news/media (9%), marketing strategies (7%), others (2%)
	Focus on individual private forest owners (52%)	Target (35%)/do not target (2%) measures, increased involvement (20%)
	Improvement of information content (22%)	Ecological arguments for conservation (11%), arguments related to cultural ecosystem services (9%), economic arguments (7%), others (9%)
(c) Relationship *Relation between stakeholders* (33%)	Improvement of interaction between stakeholders (33%)	Relation between different actors (24%), relation between forest owners (17%)

Our understanding of the recommendation categories are found in italics

Referring to the conservation procedure (Table 3b, recommendations by 65%), studies proposed to enhance the distribution of information regarding conservation (recommended by 59%). Recommendations on how to better reach forest owners ranged from the adaptation of advisory services (Urquhart 2009; Salomaa et al. 2016; Pynnönen et al. 2018) to different ways to share concrete experiences through peer-learning, or regional demonstrations (Bieling 2004; Van Gossum et al. 2005; Urquhart 2009; Korhonen et al. 2013; Joa and Schraml 2020), education (Uliczka et al. 2004; Ingemarson et al. 2006), and marketing strategies (Mäntymaa et al. 2009; Urquhart and Courtney 2011). Furthermore, a distribution of information to other relevant stakeholders such as forest advisers (Salomaa et al. 2016), the public (Jakobsson et al. 2021), or the social networks of forest owners (Vainio et al. 2018) was suggested. Moreover, an adaption of the information content was advised (recommended by 22%) as some authors regarded a stronger focus on ecological (Mäntymaa et al. 2009; Danley et al. 2021) or cultural (Paloniemi and Tikka 2008; Häyrinen et al. 2015) aspects of conservation as helpful. Additionally, economic arguments were considered relevant (Mäntymaa et al. 2009; Lindhjem and Mitani 2012; Korhonen et al. 2013). Other recommendations of the conservation procedure are focusing on individual private forest owners (recommended by 52%). While 35% of the studies recommended to target measures towards specific forest owners, it was also pointed out that this effort might not be worth the benefits. Instead, it was suggested to aim instruments at different owners’ motivations simultaneously (Danley 2019). Moreover, our core studies frequently recommended to stronger involve forest owners into the conservation process by collaboration (Brukas et al. 2018), participation (Bergseng and Vatn 2009), and consideration of local knowledge (Salomaa et al. 2016) among others.

As a third focus, we identified recommendations concerning relationships between different stakeholders (recommended by 33%, Table 3c). A third of the core studies suggested to improve the interactions between forest owners and other stakeholders (Urquhart and Courtney 2011; Eggers et al. 2014; Salomaa et al. 2016; Vainio et al. 2018) as well as amongst forest owners (Van Gossum et al. 2005; Nordén et al. 2017; Vainio et al. 2018) (recommended by 24 and 17% respectively). The described advantages of a better relation amongst the stakeholders ranged from mutual learning (Bergseng and Vatn 2009; Korhonen et al. 2013; Joa and Schraml 2020), a higher uptake (Mäntymaa et al. 2009), and an increased quality of conservation measures (Nordén et al. 2017), to reduced costs (Polomé 2016), and conflict resolution (Jakobsson et al. 2021).

## Discussion

Small-scale forest owners, which are Europe’s main forest ownership group, are important but frequently overlooked stakeholders. Acknowledging the high societal demands on forests, which are ranging from timber production, over recreation, to carbon storage, and climate change mitigation, this literature review focused on the relations between small-scale forest ownership and nature conservation. We assessed the applied research approaches, identified the social-ecological factors influencing small-scale private forest owners’ conservation perspectives, and summarized the recommendations regarding conservation policies that were derived by the core studies.

Our analysis of the state of research showed the diversity of different research approaches. We identified a trend towards quantitative studies taking place at the sub-national level. Even though our search strategy focused on smallholder or non-industrial forest owners, only a minority of the core studies investigated average forest holding sizes smaller than 20 ha. However, many authors did not specify these figures. Reasons might include the design of the research questions, the difficulty to reach these owners, or just the lack of data to calculate the average holding sizes. Apart from studies covering multiple countries, we identified a spatial focus on Sweden and Finland. Only two studies focused on conservation perspectives of small-scale forest owners in the Mediterranean region (Polomé 2016; Gatto et al. 2019) and merely one study took place in an Eastern European country (Brukas et al. 2018), if only considering studies on a sub-national or national scale.

Given the great importance of privately owned forests for biodiversity conservation in Europe, particularly with regard to the European Union’s Natura 2000 system of protected areas (European Commission 2015), we would like to encourage researchers to conduct further studies on this topic in more countries and regions. Furthermore, a subsequent synthesis of research in other regions with a high share of private forest ownership could increase mutual learning on a global scale. In addition, a review on small-scale forest ownership in former socialist countries which were only scarcely represented in our study might provide useful knowledge to complete the picture.

Private forest owners are influenced by their social-ecological environment. This latter term refers to the interplay between sociodemographic variables (Uliczka et al. 2004), structural drivers, knowledge (Deuffic et al. 2018), social context (Salomaa et al. 2016), values, and beliefs (Jokinen et al. 2018), as well as of the historical and cultural background of each country (Nijnik et al. 2016). While our literature review simplified this complexity, it allowed us to discover trends and patterns. Despite the diversity of research approaches taken, we were able to identify different key drivers that were linked to either a stronger or weaker expression of conservation perspectives. For example, being old, male, and rural-oriented had a weakening effect. Since the numbers of female (Hamunen et al. 2020) as well as non-resident or urban forest owners are increasing (Nordlund and Westin 2011; Eggers et al. 2014), there are growing opportunities to integrate nature conservation into forest management decisions. This changing ownership structure, however, is accompanied by an increase of forest owners with little knowledge and interest in forest management (Weiss et al. 2019). Such passive or uninterested owners are frequently underrepresented in social surveys (Bieling 2004) and difficult to reach but can make up large proportions amongst private forest owners (Pezdevšek Malovrh et al. 2015). Moreover, the high average age within the private forest ownership will influence current forest management practices (Schmithüsen and Hirsch 2010). It remains therefore questionable how more or even innovative nature conservation measures can be successfully implemented.

Studies elaborating the background behind forest owners’ perspectives on conservation are crucial to both understand the reasoning behind a certain variable and to identify potential approaches for improving policies (Mitani and Lindhjem 2015; Butler et al. 2016). In this regard, our review revealed that the sentimental value as a socio-ecological factor occasionally has a negative influence on conservation perspectives. Studies considering this variable pointed out that private forest owners who perceive their forest ownership as family tradition have a strong sense of place and see themselves as guardians of their forest. Therefore, they are not willing to accept restrictions on their rights and responsibilities due to additional conservation measures (Bergstén et al. 2018). However, one has to carefully consider the difference between perspectives on certain aspects of conservation policy and forest management decisions themselves. Indeed, forest structures of particular conservation concern in small-scale private forests can be related to historical forest management practices such as coppicing (Mölder 2016). While the background of this parameter is quite complex, other key factors can be described as more straightforward. For example, people who own large forest parcels have a reduced interest in nature conservation since the holding size is being closely linked to the income generated from the forest (Feliciano et al. 2017). This income might be reduced when new conservation policies become implemented. Opposed to this, a formalization of forest management positively influences conservation perspectives which might indicate that conservation measures are feasible with adequate support. However, the negative effect of economic management factors as well as of economic property values on conservation perspectives indicates that current conservation instruments rarely allow for successful integration of both conservation and resource use in private forest management.

Within this context, it is crucial to consider forestry stakeholders with a direct influence on private forest owners. Forest officers (Nordén et al. 2017) and forestry service providers (Joa and Schraml 2020) often have a rather production-oriented focus on forest management which might promote a similar focus of forest owners (Nordén et al. 2017) and limit an intrinsic interest in conservation (Joa and Schraml 2020). Similarly, a difference between the attitudes of forest officers and forest owners was found in Sweden (Kindstrand et al. 2008). As a consequence, an increasing share of forest owners is not reached by forest officers (Häyrinen et al. 2015). At the same time, the importance of forest officers and forestry service providers might grow as private forest owners are increasingly delegating forestry work and decisions to advisors (Deuffic et al. 2018), owner associations, enterprises (Eggers et al. 2014), or planning and operating organizations (Takala et al. 2019). This development might be a consequence of a changing ownership structure and result in a reduced level of skills and active decision-making (Deuffic et al. 2018). Thus, it seems to be in the interest of forest owners, forest administration, and forest service providers to stronger consider conservation aspects next to resource use.

In general, an urgent need for improved forest conservation policy can be deduced from the high share of recommendations made in all three areas of the natural resource conflict framework by Walker and Daniels (1997): substance, procedure, and relationship. Conservation instruments as well as the conservation processes need to be attractive for private forest owners with different backgrounds. Moreover, the communication of information and the interaction between the stakeholders needs to be improved. Conservation policies that consider the motivations and perspectives of private forest owners show high potential as studies indicate that high profits or a sole focus on timber production are not central to many forest owners. Instead, their aims include ecosystem-centered management (Feliciano et al. 2017), a resilient forest structure (Lupp et al. 2017), non-market objectives (Urquhart 2009), or multifunctional approaches (Hallikainen et al. 2010; Pynnönen et al. 2018). Moreover, cultural ecosystem services are important for some forest owners and influence their decision-making (Plieninger et al. 2015; Torralba et al. 2020). Especially new forest owners focus on recreation and conservation objectives (Hogl et al. 2005). It is very important, however, not to repel forest owners who are primarily interested in the use of their wood resources. Here, feasible strategies that integrate resource use and nature conservation and thus allow for successful multifunctional forest management also in small private forests are important (Kraus and Krumm 2013). For example, retention forestry accompanied by suitable legislation and incentives has the potential to complement stricter conservation approaches (Demant et al. 2020; Gustafsson et al. 2020). Given the current socio-demographic changes, it is further necessary to consider new forest owners with only a loose connection to their forests (Hogl et al. 2005). Sustaining traditional knowledge and a relation to the forest is crucial to keep them interested in forest-related issues such as nature conservation.

## Conclusions

Research approaches that analyze small-scale private forest owners and their relation to nature conservation cover a variety of thematic core areas, scales, and methods. Only a minority of studies applied qualitative methods, concentrated on small-scale forest owners (< 20 ha), had an international scope, or focused on Mediterranean or Eastern European countries. Conservation perspectives of small-scale private forest owners are crucially influenced by social-ecological drivers. We found a strengthening effect that is related to female gender, higher levels of education, formalized forest management, an active relation to the forest, and ecological values of the property. On the other hand, high age, a rural orientation, economic forest management factors, large holding size, as well as perceived economic and sentimental property value reduced the likelihood of positive perspectives towards conservation. Furthermore, recommendations regarding conservation policy were categorized using the natural resource conflict framework by Walker and Daniels (1997). Following this framework, forest conservation policy needs to be improved regarding its substance, procedure, and concerning the relationships between the stakeholders. We synthesize that the design of policy instruments needs to be stronger adapted to the forest owners, that the relevant information has to be better distributed among them, and that the interaction between different stakeholders needs to be intensified. It is especially important to include private forest owners interested in the use of their wood resources as well as owners with a loose connection to their forest. The integration of resource use and nature conservation and, thus, multifunctional approaches are crucial since forest management is confronted with increasing challenges in the face of climate change and a variety of societal demands.

## Supplementary Information

Below is the link to the electronic supplementary material.

Supplementary file1 (PDF 393KB)

## Data Availability

The data on which this review is based can be found on the platform Zenodo by using the following link https://doi.org/10.5281/zenodo.5091422.

## References

[CR1] Bergseng E, Vatn A (2009). Why protection of biodiversity creates conflict: Some evidence from the Nordic countries. Journal of Forest Economics.

[CR2] Bergstén S, Stjernström O, Pettersson Ö (2018). Experiences and emotions among private forest owners versus public interests: Why ownership matters. Land Use Policy.

[CR3] Bieling C (2004). Non-industrial private-forest owners: Possibilities for increasing adoption of close-to-nature forest management. European Journal of Forest Research.

[CR4] Bieling C, Schraml U (2004). What is closer to nature than the forest? About private owners’ perception of their forests. Allgemeine Forst- und Jagdzeitung.

[CR5] Boon TE, Meilby H (2007). Describing management attitudes to guide forest policy implementation. Small-scale Forestry.

[CR6] Borrass L, Kleinschmit D, Winkel G (2017). The “German model” of integrative multifunctional forest management: Analysing the emergence and political evolution of a forest management concept. Forest Policy and Economics.

[CR7] Bostedt G, Zabel A, Ekvall H (2019). Planning on a wider scale – Swedish forest owners’ preferences for landscape policy attributes. Forest Policy and Economics.

[CR8] Brukas V, Stanislovaitis A, Kavaliauskas M, Gaižutis A (2018). Protecting or destructing? Local perceptions of environmental consideration in Lithuanian forestry. Land Use Policy.

[CR9] Butler SM, Butler BJ, Markowski-Lindsay M (2016). Family forest owner characteristics shaped by life cycle, cohort, and period effects. Small-scale Forestry.

[CR10] Danley B (2019). Forest owner objectives typologies: Instruments for each owner type or instruments for most owner types?. Forest Policy and Economics.

[CR11] Danley B, Bjärstig T, Sandström C (2021). At the limit of volunteerism? Swedish family forest owners and two policy strategies to increase forest biodiversity. Land Use Policy.

[CR12] Demant L, Bergmeier E, Walentowski H, Meyer P (2020). Suitability of contract-based nature conservation in privately-owned forests in Germany. Nature Conservation.

[CR13] Deuffic P, Sotirov M, Arts B (2018). “Your policy, my rationale”. How individual and structural drivers influence European forest owners’ decisions. Land Use Policy.

[CR14] Edwards P, Kleinschmit D (2013). Towards a European forest policy: Conflicting courses. Forest Policy and Economics.

[CR15] Eggers J, Lämås T, Lind T, Öhman K (2014). Factors influencing the choice of management strategy among small-scale private forest owners in Sweden. Forests.

[CR16] European Commission. 2013. *Communication from the Commission to the European Parliament, the Council, the European Economic and Social Committee and the Committee of the Regions. A new EU Forest Strategy: for forests and the forest-based sector*. Brussels: European Commission.

[CR17] European Commission. 2015. *Natura 2000 and Forests. Part I-II.* Luxemburg: Publications Office of the EU.

[CR18] Feil P, Neitzel C, Seintsch B (2018). Privatwaldeigentümer in Deutschland: Ergebnisse einer bundesweiten Telefonbefragung von Personen mit und ohne Waldeigentum [Forest owners in Germany: Results of a nationwide survey of persons with and without forest property]. Applied Agricultural and Forestry Research.

[CR19] Feliciano D, Bouriaud L, Brahic E, Deuffic P, Dobsinska Z, Jarsky V, Lawrence A, Nybakk E (2017). Understanding private forest owners’ conceptualisation of forest management: Evidence from a survey in seven European countries. Journal of Rural Studies.

[CR20] Forest Europe. 2015. *State of Europe’s Forests 2015*. Madrid: Ministerial Conference on the Protection of Forests in Europe.

[CR21] Gatto P, Defrancesco E, Mozzato D, Pettenella D (2019). Are non-industrial private forest owners willing to deliver regulation ecosystem services? Insights from an alpine case. European Journal of Forest Research.

[CR22] Götmark F (2009). Conflicts in conservation: Woodland key habitats, authorities and private forest owners in Sweden. Scandinavian Journal of Forest Research.

[CR23] Gustafsson L, Bauhus J, Asbeck T, Augustynczik ALD, Basile M, Frey J, Gutzat F, Hanewinkel M (2020). Retention as an integrated biodiversity conservation approach for continuous-cover forestry in Europe. Ambio.

[CR24] Hallikainen V, Hyppönen M, Pernu L, Puoskari J (2010). Family forest owners’ opinions about forest management in northern Finland. Silva Fennica.

[CR25] Hamunen K, Muttilainen H, Tikkanen J, Hujala T (2020). Towards gender equality in family forestry: Building self-efficacy together with other female forest owners. Scandinavian Journal of Forest Research.

[CR26] Haugen K (2016). Contested lands? Dissonance and common ground in stakeholder views on forest values. Tijdschrift Voor Economische En Sociale Geografie.

[CR27] Häyrinen L, Mattila O, Berghäll S, Toppinen A (2015). Forest owners’ socio-demographic characteristics as predictors of customer value: Evidence from Finland. Small-scale Forestry.

[CR28] Hogl K, Pregernig M, Weiss G (2005). What is new about new forest owners? A typology of private forest ownership in Austria. Small-scale Forest Economics, Management and Policy.

[CR29] Hysing E, Olsson J (2005). Sustainability through good advice? Assessing the governance of Swedish forest biodiversity. Environmental Politics.

[CR30] Ingemarson F, Lindhagen A, Eriksson L (2006). A typology of small-scale private forest owners in Sweden. Scandinavian Journal of Forest Research.

[CR31] Jakobsson R, Olofsson E, Ambrose-Oji B (2021). Stakeholder perceptions, management and impacts of forestry conflicts in southern Sweden. Scandinavian Journal of Forest Research.

[CR32] Jakoby O, Lischke H, Wermelinger B (2019). Climate change alters elevational phenology patterns of the European spruce bark beetle (*Ips typographus*). Global Change Biology.

[CR33] Joa B, Schraml U (2020). Conservation practiced by private forest owners in Southwest Germany: The role of values, perceptions and local forest knowledge. Forest Policy and Economics.

[CR34] Johann F, Schaich H (2016). Land ownership affects diversity and abundance of tree microhabitats in deciduous temperate forests. Forest Ecology and Management.

[CR35] Jokinen M, Hujala T, Paloniemi R, Vainio A (2018). Private landowners and protected species: What sort of noncompliance should we be worried about?. Global Ecology and Conservation.

[CR36] Juutinen A, Tolvanen A, Koskela T (2020). Forest owners’ future intentions for forest management. Forest Policy and Economics.

[CR37] Kindstrand C, Norman J, Boman M, Mattsson L (2008). Attitudes towards various forest functions: A comparison between private forest owners and forest officers. Scandinavian Journal of Forest Research.

[CR38] Korhonen K, Hujala T, Kurttila M (2013). Diffusion of voluntary protection among family forest owners: Decision process and success factors. Forest Policy and Economics.

[CR39] Kraus, D., and F. Krumm, ed. 2013. *Integrative approaches as an opportunity for the conservation of forest biodiversity*. Joensuu: European Forest Institute.

[CR40] Lindhjem H, Mitani Y (2012). Forest owners’ willingness to accept compensation for voluntary conservation: A contingent valuation approach. Journal of Forest Economics.

[CR41] Lindner M, Maroschek M, Netherer S, Kremer A, Barbati A, Garcia-Gonzalo J, Seidl R, Delzon S (2010). Climate change impacts, adaptive capacity, and vulnerability of European forest ecosystems. Forest Ecology and Management.

[CR42] Lupp G, Börtitz K, Kantelberg V, Koch M, Pauleit S (2017). Management of urban woodlands between demands of society and owner objectives. Schweizerische Zeitschrift für Forstwesen.

[CR43] Mäntymaa E, Juutinen A, Mönkkönen M, Svento R (2009). Participation and compensation claims in voluntary forest conservation: A case of privately owned forests in Finland. Forest Policy and Economics.

[CR44] Mayer AL (2019). Family forest owners and landscape-scale interactions: A review. Landscape and Urban Planning.

[CR45] Mitani Y, Lindhjem H (2015). Forest owners’ participation in voluntary biodiversity conservation: What does it take to forgo forestry for eternity?. Land Economics.

[CR46] Moher D, Liberati A, Tetzlaff J, Altman DG, Grup TP (2009). Preferred reporting items for systematic reviews and meta-analyses: The PRISMA statement. PLOS Medicine.

[CR47] Mölder A (2016). Small forest parcels, management diversity and valuable coppice habitats: An 18th century political compromise in the Osnabrück region (NW Germany) and its long-lasting legacy. iForest.

[CR48] Mölder, A., M. Tiebel, and T. Plieninger. 2021. On the interplay of ownership patterns, biodiversity, and conservation in past and present temperate forest landscapes of Europe and North America. *Current Forestry Reports* (in press).

[CR49] Niemelä J, Young J, Alard D, Askasibar M, Henle K, Johnson R, Kurttila M, Larsson T-B (2005). Identifying, managing and monitoring conflicts between forest biodiversity conservation and other human interests in Europe. Forest Policy and Economics.

[CR50] Nijnik M, Nijnik A, Brown I (2016). Exploring the linkages between multifunctional forestry goals and the legacy of spruce plantations in Scotland. Canadian Journal of Forest Research.

[CR51] Nordén A, Coria J, Jönsson AM, Lagergren F, Lehsten V (2017). Divergence in stakeholders’ preferences: Evidence from a choice experiment on forest landscapes preferences in Sweden. Ecological Economics.

[CR52] Nordlund A, Westin K (2011). Forest values and forest management attitudes among private forest owners in Sweden. Forests.

[CR53] Paloniemi R, Tikka PM (2008). Ecological and social aspects of biodiversity conservation on private lands. Environmental Science and Policy.

[CR54] Pezdevšek Malovrh Š, Nonic D, Nedeljković J, Predrag G, Avdibegovic M, Krč J (2015). Private forest owner typologies in Slovenia and Serbia: Targeting private forest owner groups for policy implementation. Small-scale Forestry.

[CR55] Plieninger T, Bieling C, Fagerholm N, Byg A, Hartel T, Hurley P, Lopez-Santiago CA, Nagabhatla N (2015). The role of cultural ecosystem services in landscape management and planning. Current Opinion in Environmental Sustainability.

[CR56] Polomé P (2016). Private forest owners motivations for adopting biodiversity-related protection programs. Journal of Environmental Management.

[CR57] Primmer E, Paloniemi R, Similä J, Tainio A (2014). Forest owner perceptions of institutions and voluntary contracting for biodiversity conservation: Not crowding out but staying out. Ecological Economics.

[CR58] Pullin AS, Stewart GB (2006). Guidelines for systematic review in conservation and environmental management. Conservation Biology.

[CR59] Pynnönen S, Paloniemi R, Hujala T (2018). Recognizing the interest of forest owners to combine nature-oriented and economic uses of forests. Small-scale Forestry.

[CR60] Rendenieks Z, Nikodemus O, Brūmelis G (2015). The implications of stand composition, age and spatial patterns of forest regions with different ownership type for management optimisation in northern Latvia. Forest Ecology and Management.

[CR61] Richnau G, Angelstam P, Valasiuk S, Zahvoyska L, Axelsson R, Elbakidze M, Farley J, Jönsson I (2013). Multifaceted value profiles of forest owner categories in South Sweden: The river Helge å catchment as a case study. Ambio.

[CR62] Salomaa A, Paloniemi R, Hujala T, Rantala S, Arponen A, Niemelä J (2016). The use of knowledge in evidence-informed voluntary conservation of Finnish forests. Forest Policy and Economics.

[CR63] Sandström C, Carlsson-Kanyama A, Lindahl KB, Sonnek KM, Mossing A, Nordin A, Nordström E-M, Räty R (2016). Understanding consistencies and gaps between desired forest futures: An analysis of visions from stakeholder groups in Sweden. Ambio.

[CR64] Schaich H, Plieninger T (2013). Land ownership drives stand structure and carbon storage of deciduous temperate forests. Forest Ecology and Management.

[CR65] Schmithüsen F, Hirsch F (2010). Private forest ownership in Europe. Geneva Timber and Forest Study Papers.

[CR66] Seidl R, Rammer W, Jäger D, Currie WS, Lexer MJ (2007). Assessing trade-offs between carbon sequestration and timber production within a framework of multi-purpose forestry in Austria. Forest Ecology and Management.

[CR67] Takala T, Hujala T, Tanskanen M, Tikkanen J (2019). Competing discourses of the forest shape forest owners’ ideas about nature and biodiversity conservation. Biodiversity and Conservation.

[CR68] Torralba M, Lovrić M, Roux J-L, Budniok M-A, Mulier A-S, Winkel G, Plieninger T (2020). Examining the relevance of cultural ecosystem services in forest management in Europe. Ecology and Society.

[CR69] Uliczka H, Angelstam P, Jansson G, Bro A (2004). Non-industrial private forest owners’ knowledge of and attitudes towards nature conservation. Scandinavian Journal of Forest Research.

[CR70] UNECE, FAO (2020). Who owns our forests? Forest ownership in the ECE region.

[CR71] Urquhart, J. 2009. Public good delivery in private woodlands in England: An empirically-based typology of small-scale private forest owners. In *Seeing the forest beyond the trees. New possibilities and expectations for products and services from small-scale forestry. Proceedings of the 2009 IUFRO 3.08 Small-Scale Forestry Symposium*, pp. 255–269. Morgantown, West Virginia (USA).

[CR72] Urquhart J, Courtney P (2011). Seeing the owner behind the trees: A typology of small-scale private woodland owners in England. Forest Policy and Economics.

[CR73] Urquhart J, Courtney P, Slee B (2012). Private woodland owners’ perspectives on multifunctionality in English woodlands. Journal of Rural Studies.

[CR74] Vainio A, Paloniemi R, Hujala T (2018). How are forest owners’ objectives and social networks related to successful conservation?. Journal of Rural Studies.

[CR75] Van Gossum P, Luyssaert S, Serbruyns I, Mortier F (2005). Forest groups as support to private forest owners in developing close-to-nature management. Forest Policy and Economics.

[CR76] Van Gossum P, Arts B, De Wulf R, Verheyen K (2011). An institutional evaluation of sustainable forest management in Flanders. Land Use Policy.

[CR77] Vedel SE, Jacobsen JB, Thorsen BJ (2015). Forest owners’ willingness to accept contracts for ecosystem service provision is sensitive to additionality. Ecological Economics.

[CR78] Walker, G.B., and S.E. Daniels. 1997. Foundations of natural resource conflict. In *Conflict management and public participation in land management*, pp. 13–36. EFI Proceedings 14. Joensuu: European Forest Institute.

[CR79] Weiss G, Lawrence A, Hujala T, Lidestav G, Nichiforel L, Nybakk E, Quiroga S, Sarvašová Z (2019). Forest ownership changes in Europe: State of knowledge and conceptual foundations. Forest Policy and Economics.

[CR80] Widman U (2015). Shared responsibility for forest protection?. Forest Policy and Economics.

[CR81] Wiersum KF, Elands BHM, Hoogstra MA (2005). Small-scale forest ownership across Europe: Characteristics and future potential. Small-scale Forest Economics, Management and Policy.

[CR82] Wilkes-Allemann, J., and E. Lieberherr. 2020. Implications of forest ownership changes for forest and biodiversity governance and management. In *How to balance forestry and biodiversity conservation. A view across Europe*, ed. F. Krumm, A. Schuck, and A. Rigling, 77–86. Birmensdorf: European Forest Institute (EFI); Swiss Federal Institute for Forest, Snow and Landscape Research (WSL).

[CR83] Wulf M, Kolk J (2014). Plant species richness of very small forests related to patch configuration, quality, heterogeneity and history. Journal of Vegetation Science.

[CR84] Zivojinovic, I., G. Weiss, G. Lidestav, D. Feliciano, Z. Dobšinská, A. Lawrence, T. Hujala, E. Nybakk, et al. (eds.). 2015. *Forest land ownership change in Europe. COST action FP1201 FACESMAP country reports, joint volume*. Vienna: University of Natural Resources and Life Sciences.

