# Supplementary material for: Conservation perspectives of small-scale private forest owners in Europe: A systematic review
Source: Ambio. 2021 Sep 20;51(4):836–48. doi: 10.1007/s13280-021-01615-w (PMC8847644; doi:10.1007/s13280-021-01615-w)
Supplement: Supplementary file 1 — Supplementary file1 (PDF 393KB) [file 13280_2021_1615_MOESM1_ESM.pdf]

Ambio: Electronic Supplementary Material

This supplementary material has not been peer reviewed.

Title: Conservation perspectives of small-scale private forest owners in Europe: A systematic review

Appendix S1: Core studies analyzed in the systematic literature review

Bergseng, E., and A. Vatn. 2009. Why protection of biodiversity creates conflict - Some evidence from the Nordic countries. *Journal of Forest Economics* 15: 147–165. doi:10.1016/j.jfe.2008.04.002.

Bergstén, S., O. Stjernström, and Ö. Pettersson. 2018. Experiences and emotions among private forest owners versus public interests: Why ownership matters. *Land Use Policy* 79: 801–811. doi:10.1016/j.landusepol.2018.08.027.

Bieling, C. 2004. Non-industrial private-forest owners: Possibilities for increasing adoption of close-to-nature forest management. *European Journal of Forest Research* 123: 293–303. doi:10.1007/s10342-004-0042-6.

Bieling, V. C., and U. Schraml. 2004. What is closer to nature than the forest? About private owners' perception of their forests. *Allgemeine Forst und Jagdzeitung* 175: 41–48.

Boon, T. E., and H. Meilby. 2007. Describing management attitudes to guide forest policy implementation. *Small-scale Forestry* 6: 79–92. doi:10.1007/s11842-007-9006-2.

Bostedt, G., A. Zabel, and H. Ekvall. 2019. Planning on a wider scale - Swedish forest owners' preferences for landscape policy attributes. *Forest Policy and Economics* 104: 170–181. doi:10.1016/j.forpol.2019.04.013.

Brukas, V., A. Stanislovaitis, M. Kavaliauskas, and A. Gaižutis. 2018. Protecting or destructing? Local perceptions of environmental consideration in Lithuanian forestry. *Land Use Policy* 79: 1014–1023. doi:10.1016/j.landusepol.2016.05.006.

Danley, B. 2018. Skepticism of state action in forest certification and voluntary set-asides: a Swedish example with two environmental offsetting options. *Scandinavian Journal of Forest Research* 33: 695–707. doi:10.1080/02827581.2018.1479442.

Danley, B. 2019. Forest owner objectives typologies: Instruments for each owner type or instruments for most owner types? *Forest Policy and Economics* 105: 72–82. doi:10.1016/j.forpol.2019.05.018.

Danley, B., T. Bjärstig, and C. Sandström. 2021. At the limit of volunteerism? Swedish family forest owners and two policy strategies to increase forest biodiversity. *Land Use Policy* 105: 105403. doi:10.1016/j.landusepol.2021.105403.

Deuffic, P., M. Sotirov, and B. Arts. 2018. "Your policy, my rationale". How individual and structural drivers influence European forest owners' decisions. *Land Use Policy* 79: 1024–1038. doi:10.1016/j.landusepol.2016.09.021.

Eggers, J., T. Lämås, T. Lind, and K. Öhman. 2014. Factors influencing the choice of management strategy among small-scale private forest owners in Sweden. *Forests* 5: 1695–1716. doi:10.3390/f5071695.

Eriksson, L., and C. Fries. 2020. The Knowledge and Value Basis of Private Forest Management in Sweden: Actual Knowledge, Confidence, and Value Priorities. *Environmental Management* 66: 549–563. doi:10.1007/s00267-020-01328-y.

Feliciano, D., L. Bouriaud, E. Brahic, P. Deuffic, Z. Dobsinska, V. Jarsky, A. Lawrence, E. Nybakk, et al. 2017. Understanding private forest owners' conceptualisation of forest management: Evidence from a survey in seven European countries. *Journal of Rural Studies* 54: 162–176. doi:10.1016/j.jrurstud.2017.06.016.

Gatto, P., E. Defrancesco, D. Mozzato, and D. Pettenella. 2019. Are non-industrial private forest owners willing to deliver regulation ecosystem services? Insights from an alpine case. *European Journal of Forest Research* 138: 639–651. doi:10.1007/s10342-019-01195-1.

Hallikainen, V., M. Hyppönen, L. Pernu, and J. Puoskari. 2010. Family forest owners' opinions about forest management in northern Finland. *Silva Fennica* 44: 363–384. doi:10.14214/sf.158.

Hayrinen, L., O. Mattila, S. Berghall, and A. Toppinen. 2015. Forest owners' socio-demographic characteristics as predictors of customer value: Evidence from Finland. *Small-Scale Forestry* 14: 19–37. doi:10.1007/s11842-014-9271-9.

Hysing, E., and J. Olsson. 2005. Sustainability through good advice? Assessing the governance of Swedish forest biodiversity. *Environmental Politics* 14: 510–526. doi:10.1080/09644010500175742.

Ingemarson, F., A. Lindhagen, and L. Eriksson. 2006. A typology of small-scale private forest owners in Sweden. *Scandinavian Journal of Forest Research* 21: 249–259. doi:10.1080/02827580600662256.

Jakobsson, R., E. Olofsson, and B. Ambrose-Oji. 2021. Stakeholder perceptions, management and impacts of forestry conflicts in southern Sweden. *Scandinavian Journal of Forest Research* 36: 68–82. doi:10.1080/02827581.2020.1854341.

Joa, B., and U. Schraml. 2020. Conservation practiced by private forest owners in Southwest Germany – The role of values, perceptions and local forest knowledge. *Forest Policy and Economics* 115: 102141. doi:10.1016/j.forpol.2020.102141.

Jokinen, M., T. Hujala, R. Paloniemi, and A. Vainio. 2018. Private landowners and protected species: What sort of noncompliance should we be worried about? *Global Ecology and Conservation* 15: e00407. doi:10.1016/j.gecco.2018.e00407.

Juutinen, A., A. Tolvanen, and T. Koskela. 2020. Forest owners' future intentions for forest management. *Forest Policy and Economics* 118. doi:10.1016/j.forpol.2020.102220.

Korhonen, K., T. Hujala, and M. Kurttila. 2013. Diffusion of voluntary protection among family forest owners: Decision process and success factors. *Forest Policy and Economics* 26: 82–90. doi:10.1016/j.forpol.2012.08.010.

Koskela, T., and H. Karppinen. 2021. Forest owners' willingness to implement measures to safeguard biodiversity: Values, attitudes, ecological worldview and forest ownership objectives. *Small-Scale Forestry* 20: 11–37. doi:10.1007/s11842-020-09454-5.

Lindhjem, H., and Y. Mitani. 2012. Forest owners' willingness to accept compensation for voluntary conservation: A contingent valuation approach. *Journal of Forest Economics* 18: 290–302. doi:10.1016/j.jfe.2012.06.004.

Mäntymaa, E., A. Juutinen, M. Mönkkönen, and R. Svento. 2009. Participation and compensation claims in voluntary forest conservation: A case of privately owned forests in Finland. *Forest Policy and Economics* 11: 498–507. doi:10.1016/j.forpol.2009.05.007.

Mitani, Y., and H. Lindhjem. 2015. Forest owners' participation in voluntary biodiversity conservation: What does it take to forgo forestry for eternity? *Land Economics* 91: 235–251. doi:10.3368/le.91.2.235.

Nordén, A., J. Coria, A. M. Jönsson, F. Lagergren, and V. Lehsten. 2017. Divergence in stakeholders' preferences: Evidence from a choice experiment on forest landscapes preferences in Sweden. *Ecological Economics* 132: 179–195. doi:10.1016/j.ecolecon.2016.09.032.

Nordlund, A., and K. Westin. 2011. Forest values and forest management attitudes among private forest owners in Sweden. *Forests* 2: 30–50. doi:10.3390/f2010030.

Paloniemi, R., and P. M. Tikka. 2008. Ecological and social aspects of biodiversity conservation on private lands. *Environmental Science and Policy* 11: 336–346. doi:10.1016/j.envsci.2007.11.001.

Polomé, P. 2016. Private forest owners motivations for adopting biodiversity-related protection programs. *Journal of Environmental Management* 183: 212–219. doi:10.1016/j.jenvman.2016.07.097.

Primmer, E., R. Paloniemi, J. Similä, and A. Tainio. 2014. Forest owner perceptions of institutions and voluntary contracting for biodiversity conservation: Not crowding out but staying out. *Ecological Economics* 103: 1–10. doi:10.1016/j.ecolecon.2014.04.008.

Pynnönen, S., R. Paloniemi, and T. Hujala. 2018. Recognizing the interest of forest owners to combine nature-oriented and economic uses of forests. *Small-Scale Forestry* 17: 443–470. doi:10.1007/s11842-018-9397-2.

Salomaa, A., R. Paloniemi, T. Hujala, S. Rantala, A. Arponen, and J. Niemelä. 2016. The use of knowledge in evidence-informed voluntary conservation of Finnish forests. *Forest Policy and Economics* 73: 90–98. doi:10.1016/j.forpol.2016.09.004.

Takala, T., T. Hujala, M. Tanskanen, and J. Tikkanen. 2019. Competing discourses of the forest shape forest owners' ideas about nature and biodiversity conservation. Biodiversity and Conservation 28: 3445–3464. doi:10.1007/s10531-019-01831-7.

Uliczka, H., P. Angelstam, G. Jansson, and A. Bro. 2004. Non-industrial private forest owners' knowledge of and attitudes towards nature conservation. *Scandinavian Journal of Forest Research* 19: 274–288. doi:10.1080/02827580410029318.

Urquhart, J. 2009. Public good delivery in private woodlands in England: An empirically-based typology of small-scale private forest owners. In *Seeing the forest beyond the trees. New possibilities and expectations for products and services from small-scale forestry*, 353. Morgantown, West Virginia (USA).

Urquhart, J., and P. Courtney. 2011. Seeing the owner behind the trees: A typology of small-scale private woodland owners in England. *Forest Policy and Economics* 13: 535–544. doi:10.1016/j.forpol.2011.05.010.

Urquhart, J., P. Courtney, and B. Slee. 2012. Private woodland owners' perspectives on multifunctionality in English woodlands. *Journal of Rural Studies* 28: 95–106. doi:10.1016/j.jrurstud.2011.08.006.

Vainio, A., R. Paloniemi, and T. Hujala. 2018. How are forest owners' objectives and social networks related to successful conservation? *Journal of Rural Studies* 62: 21–28. doi:10.1016/j.jrurstud.2018.06.009.

Van Gossum, P., S. Luyssaert, I. Serbruyns, and F. Mortier. 2005. Forest groups as support to private forest owners in developing close-to-nature management. *Forest Policy and Economics* 7: 589–601. doi:10.1016/j.forpol.2003.10.003.

Van Gossum, P., B. Arts, R. De Wulf, and K. Verheyen. 2011. An institutional evaluation of sustainable forest management in Flanders. *Land Use Policy* 28: 110–123. doi:10.1016/j.landusepol.2010.05.005.

Vedel, S. E., J. B. Jacobsen, and B. J. Thorsen. 2015. Forest owners' willingness to accept contracts for ecosystem service provision is sensitive to additionality. *Ecological Economics* 113: 15–24. doi:10.1016/j.ecolecon.2015.02.014.

Widman, U. 2015. Shared responsibility for forest protection? *Forest Policy and Economics* 50: 220–227. doi:10.1016/j.forpol.2014.10.003.

Wiersum, K. F., B. H. M. Elands, and M. A. Hoogstra. 2005. Small-scale forest ownership across Europe: Characteristics and future potential. *Small-scale Forest Economics, Management and Policy* 4: 1–19. doi:10.1007/s11842-005-0001-1.
